# Glucose Oxidase Initiates Radical Polymerizations by Direct Electron Transfer to Monomers

**DOI:** 10.1021/acs.biomac.5c01372

**Published:** 2025-09-25

**Authors:** Eleonora Ornati, Iuliia Ushakova, Nico Bruns

**Affiliations:** 1 Department of Chemistry and Centre for Synthetic Biology, 26536 Technical University of Darmstadt, Peter-Grünberg-Str. 4, 64287 Darmstadt, Germany; 2 Department of Pure and Applied Chemistry, University of Strathclyde, Thomas Graham House, 295 Cathedral Street, Glasgow G1 1XL, U.K.

## Abstract

Glucose oxidase (GOx) is a widely used and studied enzyme, yet it continues to surprise with previously unknown activities. We report GOx to initiate radical polymerizations of acrylamides and methacrylates without the need for initiators or irradiation by light, simply by carrying out the polymerizations in the absence of oxygen at high glucose concentrations. The enzyme oxidizes glucose and concomitantly transfers an electron and a proton to a monomer, thereby creating a radical species that starts the polymerization. Computational docking studies revealed specific orientations of monomers in the enzyme’s active site. GOx’s ability to deoxygenate solutions was combined with its initiation activity to achieve polymerizations in nondeoxygenated conditions, allowing polymerizations in a 96-well plate format, and a fluorescence assay was developed to screen the enzyme’s polymerization activity. GOx’s polymerization activity opens the route to polymer synthesis under mild and biological relevant conditions and allows integration of GOx-catalyzed radical polymerizations into biosensors as well as living and synthetic cells.

## Graphical abstract

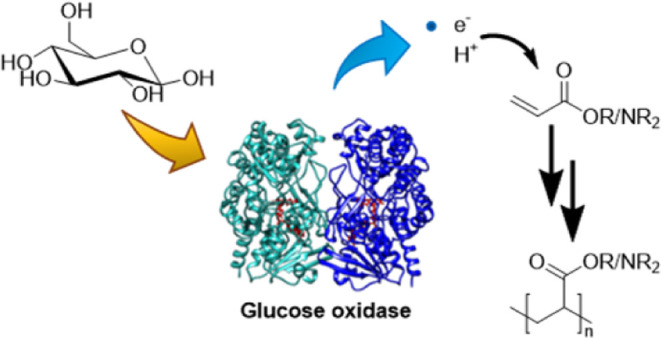

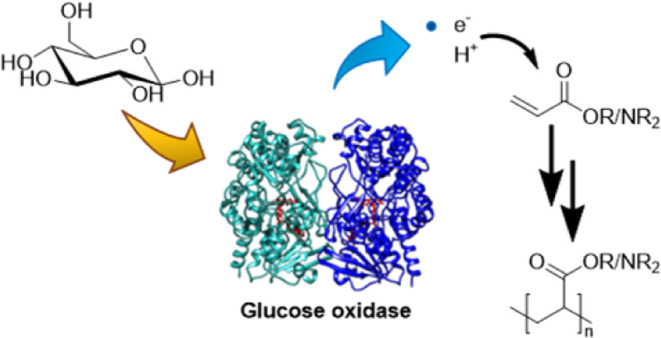

## Introduction

Protein-mediated and enzyme-catalyzed radical polymerizations offer a sustainable approach for polymer synthesis under mild conditions.^1–7^ They bridge the synthetic with the natural world, enabling applications such as the engineering of living cells with synthetic polymers,^8–12^ preparing synthetic cells,^13^ and diagnosing^14,15^ or treating diseases.^8,16^ Such polymerizations have been successfully demonstrated with various proteins and enzymes, revealing the hidden potential of biological catalysts to synthesize polymers such as polymethacrylates, polyacrylates, and polyacrylamides.^1–7^ For example, xanthine oxidase^17^ and horseradish peroxidase^18,19^ have long been known to initiate free radical polymerizations. Peroxidases and other metalloenzymes can catalyze atom transfer radical polymerizations (ATRP),^20–28^ metalloenzymes have been used to initiate reversible-addition–fragmentation chain-transfer (RAFT) polymerizations,^20,29–32^ various proteins can be used to initiate polymerizations under violet or blue light irradiation, presumably through a proton-coupled electron-transfer mechanism involving tyrosines,^33^ and glucose oxidase (GOx) has been used to initiate free radical polymerizations and RAFT polymerizations.^34–37^ GOx is a widely studied flavoprotein of the fungal glucose–methanol–choline (GMC) oxidoreductase superfamily.^38^ The enzyme is responsible for the oxidation of β-d-glucose and concomitant reduction of molecular oxygen via a ping-pong bibi mechanism in two steps that involve the transfer of two protons and two electrons from glucose to dioxygen through a flavin adenine dinucleotide (FAD) cofactor.^39–41^ The products of the reaction are hydrogen peroxide and d-glucono-δ-lactone. The latter spontaneously reacts with water to produce gluconic acid, which acidifies the solution, while the first can inhibit GOx activity at a high concentration.^42^ Numerous substrates have been proven to be accepted by GOx, both as electron donors and electron acceptor species.^41–44^ While the reduction of the cofactor FAD to FADH_2_ by glucose happens through a base-catalyzed hydride transfer, the oxidation of the cofactor follows a stepwise electron transfer to the O_2_ that involves the transfer of one electron at a time and the transfer of two protons to form hydrogen peroxide.^39,41,45^ GOx can also reduce various molecules through a single electron transfer, coupled or not coupled with the transfer of a proton to the molecule, depending on the nature of the acceptor.^39,44,46,47^ Various xenobiotic compounds have been proven to be suitable as direct hydrogen atom acceptors from the flavoprotein,^43^ making the protein a versatile enzyme for different reactivities. Thanks to its stability and reliable activity, the enzyme is widely used, e.g., in glucose sensors for medical applications^48–50^ and in the food and drink industries.^41,51,52^ Moreover, it is used in the food industry,^40,41^ e.g., to enhance the dough in bread making^53^ or to remove glucose in dry egg powder production.^54^ Glucose oxidase has also been extensively used in polymer chemistry as an oxygen scavenger system to lower the oxygen concentration in open-air vessels,^34,37,55–59^ allowing radical polymerization reaction to proceed, which otherwise would be inhibited or retarded by oxygen. Moreover, its catalytic product, hydrogen peroxide, has been used to start the radical polymerization of various monomers upon reaction with transition metals or other metal-containing proteins, such as horseradish peroxidase, to produce the starting radical species.^60–66^ GOx has also been found to initiate RAFT^36,37^ and ATRP^67^ polymerizations under violet light irradiation. Another example for GOx-mediated polymerizations is, under oxygen-free conditions, the electron transfer from the reduced cofactor to *N*-hydroxyimide compounds that then act as initiators for free radical polymerizations of acrylates^68^ and acrylamides.^69^ Thus, in the previous works, the initiation of radical polymerizations by GOx relied on either the formation of hydrogen peroxide, on the irradiation of the protein with light of relatively high energy (violet or UV), or on the direct reduction of compounds that act as initiators.

Here, we show that GOx from *Aspergillus niger* (*A. niger*) directly induces the radical polymerization of various monomers without the need of light irradiation or an additional initiator (Figure 1). Through the oxidation of glucose, the enzyme efficiently triggers initiator-free polymerizations both in anoxic, i.e., oxygen-free, conditions and in the presence of oxygen, via a single electron transfer combined with proton transfer to the monomer. These findings not only enrich the knowledge on this industrially and medically relevant oxidoreductase but also enhance the understanding of flavoproteins as catalysts for polymerization reactions. Moreover, this work establishes a biocatalytic polymerization system that can be useful for polymer synthesis, for polymerization-amplified signal detection in glucose biosensors, and to implement radical polymerizations into living cells, artificial cells, and synthetic biology systems.

**Figure 1:**
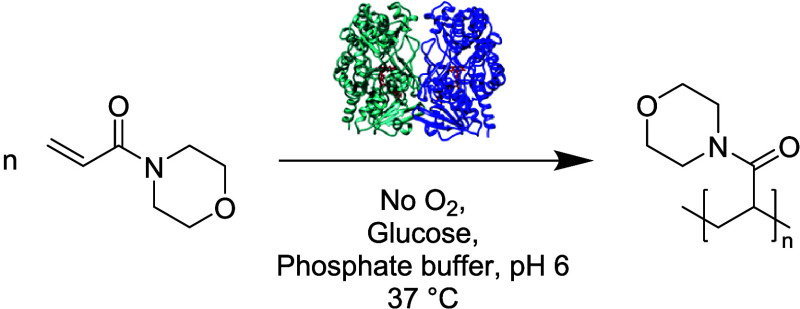
Reaction scheme of the GOx-initiated radical polymerization of, as an example, *N*-acryloylmorpholine by electron and proton transfer to the monomer, with glucose as a reducing agent for the enzyme. The enzymatic polymerization also works with other acrylamides, methacrylates, and styrene derivatives.

## Result and Discussion

### GOx-Induced Polymerization of Various Monomers

Based on our previous work on bioATRP,^11,13,22–25^ we initially tested if GOx might reduce an ATRP initiator, such as the widely used 2-hydroxyethyl 2-bromoisobutyrate (HEBIB), which would then start the polymerization of water-soluble monomers, such as *N*-isopropylacrylamide (NIPAm). A first set of reactions using 1 mg/mL GOx as a catalyst, 200 mM glucose as reducing agent for the protein, and 300 mM of NIPAm, with and without 15 mM of HEBIB, was carried out in an anoxic environment (Figure S1), established by prolonged purging of the reaction mixture with argon, and without irradiation by light. During parallel experiments, the polymerization of NIPAm at 37 °C proceeded equally in the presence or absence of the initiator but did not proceed at all when the protein was in the higher oxidation state, i.e., in the absence of glucose, or in the absence of the enzyme (Table S1). A reduced form of the enzyme, without adding the initiator, was enough to achieve 30% monomer conversion in 50 mM phosphate buffer (PB) pH 7.4, opening the hypothesis of an enzymatic activity on the monomer itself. It should be noted that, in contrast to the photoenzymatic GOx-mediated radical polymerizations reported in the literature,^36,37^ a higher glucose concentration was used in our studies. The Michaelis constant *K*_m_ for GOx from *A. niger* for the oxidation of β-d-glucose ranges from 33 to 110 mM.^70,71^ In the present study, the concentration of glucose above the *K*_m_ of the enzyme, as well as the higher concentration of GOx in the reaction mixture, might explain why the catalyst is able to initiate polymerizations by direct electron transfer to the monomer in the dark. Moreover, by avoiding light irradiation and by the control experiments provided below, there was no risk that the polymerizations got initiated by light irradiation of traces of H_2_O_2_.

Apart from NIPAm, the enzyme polymerized many other acrylamide and methacrylate monomers in the absence of the initiator and without the need to irradiate the reaction mixture with light. Acrylamide (AAm), *N*-acryloylmorpholine (NAM), 2-hydroxyethyl methacrylate (HEMA), and poly­(ethylene glycol) methacrylate (PEGMA) were all successfully polymerized after reacting overnight at 37 °C under anoxic condition (Table S2), and no polymer was found by NMR spectroscopy for the reactions performed without the enzyme or glucose (Figure S2).

The polymerization reaction was then optimized to achieve higher monomer conversion (Figure 1). GOx (to yield a final concentration of 5 mg/mL) was degassed as a powder for 1 h, while a solution of 500 mM of NAM and 200 mM of glucose in 50 mM PB pH 6 was bubbled with argon for the same amount of time. The overnight reaction yields a conversion of above 90%, with polymers of high molecular weight (*M*_n_ usually between 1 × 10^5^ and 5 × 10^5^ g/mol) and broad polydispersity (usually between 2 and 2.8), pointing toward a free radical nature of the polymerization.

Glucose oxidase produces hydrogen peroxide in the presence of molecular oxygen. Hydrogen peroxide is a reactive compound that can generate radicals under certain conditions, including light exposure and the presence of transition metal ions.^72–74^ To rule out the possibility of H_2_O_2_ being produced during the anoxic reaction of GOx with glucose and the monomers, and H_2_O_2_ being the actual source of radicals for the reaction to start, control experiments were carried out by adding H_2_O_2_ to a mixture of 500 mM of NAM in the presence of 5 mg/mL GOx or 200 mM glucose under anoxic condition. Based on the O_2_ average concentration in water and water-based buffers,^75,76^ a concentration of 1 M H_2_O_2_, was chosen for the reaction, which is far above the concentration range of dissolved oxygen in water. However, none of the conditions tested led to the polymers being produced after the overnight reaction at 37 °C (Figure S3).

The optimized anoxic polymerization of NAM with GOx as a catalyst and glucose as an electron donor for the enzyme was then repeated in the presence of 200 mM sodium pyruvate (SP). This well-known weak reducing agent can readily react with the H_2_O_2_ in solution to form acetate, water, and CO_2_,^77,78^ thus promptly removing H_2_O_2_ and preventing hydroxyl and hydroperoxyl radicals from forming. The addition of SP did not significantly change the polymerization’s success, thus ruling out the possibility that residual hydrogen peroxide caused the start of the polymerization (Figure 2a). When H_2_O_2_ was used instead of glucose or GOx, all SP got decarboxylated to form acetate, as shown by the total absence of the ^1^H NMR peak of SP at 2.3 ppm and the presence of the acetate peak at 2 ppm. Still, no polymer was detected, as shown by the absence of the typical polymer backbone peaks at around 1.5 and 2.5 ppm (Figure S3). Final control experiments were conducted by replacing GOx with a heat-denatured enzyme or by replacing glucose with sodium pyruvate, and no polymer was produced under those conditions either (Figure 2a). To exclude any contaminants in the commercial GOx lyophilized powder, which could somehow start the polymerization, commercial GOx was purified twice. First, a 15 mg/mL solution of GOx was passed through a Sephacryl S-300 HR size exclusion column in a ÄKTA pure protein purification system (Figure S4) and then through a Sephadex PD10 desalting column. The resulting purified protein was allowed to react with 500 mM NAM under anaerobic conditions, with or without SP, and conversion above 90% was reached (Figure 2a). These results suggest a catalytic activity of GOx for various water-soluble acrylamide- and methacrylate-based monomers, which presumably get reduced to form free radical species that would then start the polymerization reaction.

**Figure 2:**
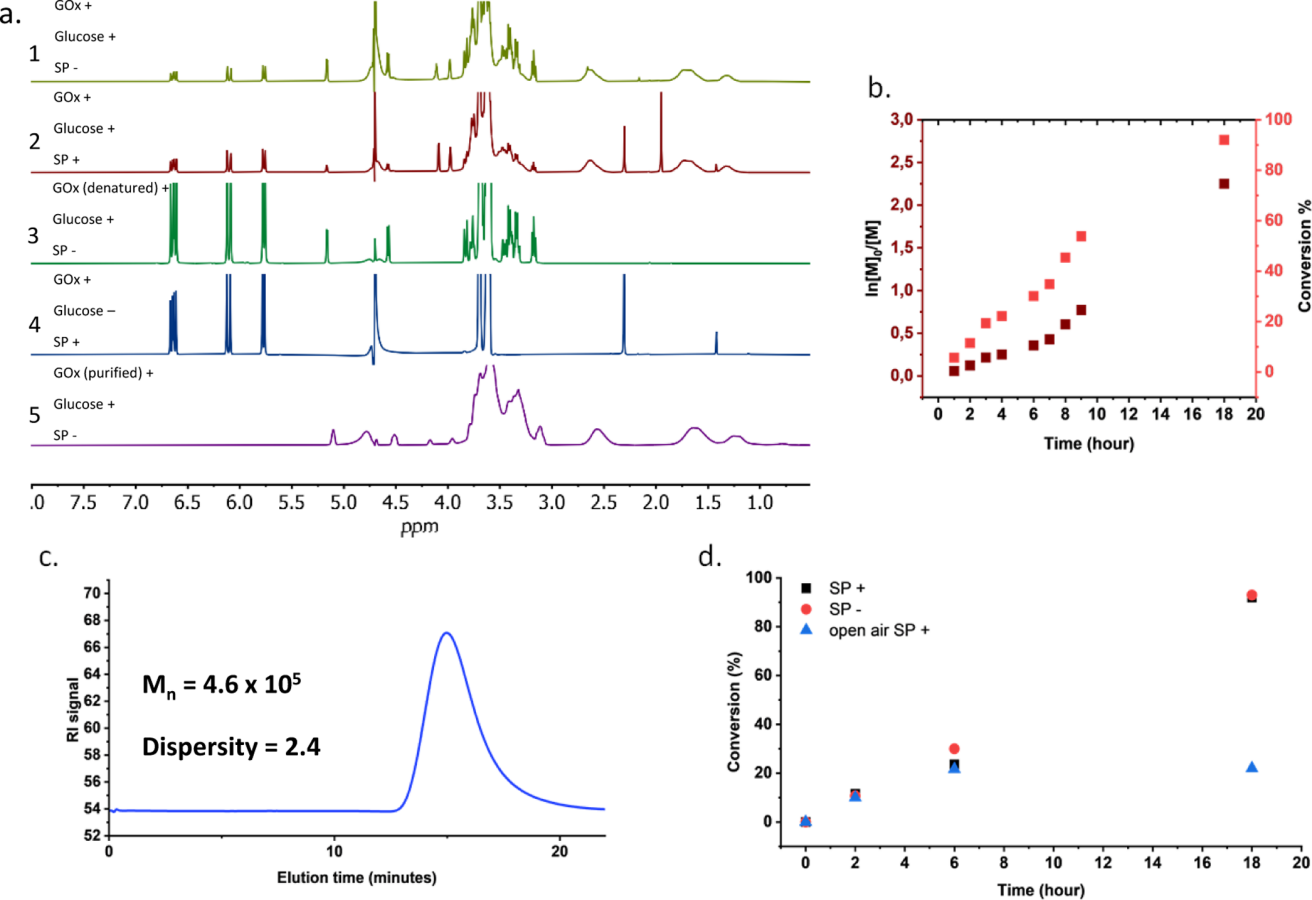
GOx-initiated polymerizations of 500 mM NAM with 5 mg/mL GOx, 200 mM glucose in anoxic condition for 18 h at 37 °C in 50 mM PB pH 6. (a) Comparison of ^1^H NMR spectra of the anoxic polymerization of NAM with GOx and glucose (1), the same reaction of (1) but with the addition of 200 mM SP (2), control reaction with denatured enzyme (3), control reaction with SP used instead of glucose (4), and purified GOx (5). PolyNAM formed when native GOx and glucose were present, but not when either of them was absent in the mix, and the presence of SP did not prevent the reaction from happening, indicating that the polymerization was not caused by H_2_O_2_. (b) Kinetics of a reaction of 5 mg/mL GOx, 200 mM glucose, and 500 mM NAM under anoxic conditions. (c) GPC elugram of polyNAM from the final time point of the anoxic reaction without SP reported in panel (d). (d) Comparison of polymerization kinetics of 5 mg/mL GOx, 200 mM glucose, and 500 mM NAM in three different conditions: anoxic reaction with and without SP, and open-air reaction with SP.

### Polymerization Kinetics

The kinetics of the GOx-initiated polymerization of NAM were then studied. The growing polymer chains were quenched by precipitating the sample in a solution of 100 mM hydroquinone in D_2_O. They were then solubilized in DMSO-d_6_ for NMR analysis. The polymerization of NAM proceeded slowly but linearly over the first 9 h, with a 50% conversion achieved around 8.5 h of reaction (Figure 2b) and reached over 90% of monomer conversion after overnight reaction. The reaction produced high-molecular-weight polymers of number-average molecular weight (*M*_n_) above 1 × 10^5^ g/mol and high *Đ* value (*Đ* > 2) at any time point analyzed, confirming the free radical mechanisms (Figure 2c and Table S3). In line with the results presented above, the presence of SP in anoxic conditions did not produce any significant changes in the polymerization kinetics, leading to a high final yield in the same amount of time and ruling out the possibility of H_2_O_2_ involvement in the process (Figure 2d and Table S3).

### Polymerization Reaction in the Open Air

Glucose oxidase can reduce the oxygen concentration enough to allow polymerization reactions to proceed in reaction vessels that are open to air.^34,55–58,66^ We therefore decided to test whether open-air polymerization could be possible when GOx was employed as both an oxygen scavenger and a direct source of radicals. GOx-catalyzed polymerization of NAM was thus repeated in fully open-air conditions. To this end, different reaction conditions were tested simultaneously in a 96-well plate in a final volume of 200 μL with 5 mg/mL GOx, 500 mM NAM, and glucose concentrations between 200 mM and 1 M. The reactions were carried out in 50 mM PB, pH 6, in the dark at 37 °C for 18 h (Table 1). The open-air reactions did not proceed well under any of the tested conditions. Practically no conversion was achieved when a mixture of GOx, glucose, and monomer was used. This may be caused by the low pH reached during the reaction (pH < 2, measured at the end of the reaction) due to the formation of gluconic acid and/or the accumulation of H_2_O_2_ in the solution. The loss in activity can be seen by the residual glucose detectable in ^1^H NMR spectra, indicating that the protein stopped working before glucose was fully consumed (Figure S5). The use of the enzyme catalase, known to efficiently remove H_2_O_2_ by converting two molecules of hydrogen peroxide into two molecules of water and one molecule oxygen, slightly increased the yield when high concentrations of glucose were used (Table 1), probably by reducing the inhibitory effect of H_2_O_2_ on GOx. This can be inferred from the higher glucose consumption during the reaction (Figure S6).

**Table 1:** Results of GOx-Initiated Polymerizations of NAM in the Presence of Oxygen^a^

	glucose 1 M					glucose 500 mM			glucose 200 mM			
	–	0.2 mg/mL catalase	200 mM SP	400 mM SP	–	0.2 mg/mL catalase	200 mM SP	400 mM SP	–	0.2 mg/mL catalase	200 mM SP	400 mM SP
Open Air												
pH at the end of the reaction	<2	<2	5	6	<2	<2	5	5	<2	<2	5	6
conversion [%]	<1	7	33	33	<1	7	31	25	<1	1	20	11
FAD oxidation state at the end of the reaction	Ox.	Ox.	Ox.	Ox.	Ox.	Ox.	Ox.	Ox.	Ox.	Ox.	Ox.	Ox.
Reduced O_2_												
pH at the end of the reaction	<2	<2	6	6	<2	<2	6	6	<2	<2	6	6
conversion [%]	49	84	91	89	44	82	91	85	33	74	92	88
FAD oxidation state at the end of the reaction	Ox.	Ox.	Red.	Red.	Ox.	Ox.	Red.	Red.	Ox.	Ox.	Red.	Red.

a Reaction conditions: 5 mg/mL GOx and 500 mM NAM. Reactions were performed in 96-well plates at 37 °C in the dark, with the wells either open to air or covered with a layer of mineral oil to achieve reduced O_2_ conditions. The reaction time was 18 h. The FAD state at the end of the reaction was determined by analyzing the absorption spectrum of the solution in the region between 400 and 500 nm where FAD and FADH_2_ can be distinguish by the presence or absence of the peak with maxima at 450 nm, respectively.^79^

Finally, SP was used at two different concentrations, namely, 200 and 400 mM. Under these conditions, the final yield of the polymerization reaction improved, bringing the conversion to around 30% after an overnight reaction (Table 1). When 200 mM SP and 200 mM glucose were used, the kinetics of the first 6 h followed the same trend as the kinetics of the same reaction in anoxic conditions (Figure 2d). However, the conversion stopped increasing after that and remained at around 20%, probably marking the time glucose finished or SP concentrations were too low to avoid H_2_O_2_ accumulation and GOx inhibition. SP might be beneficial to the reaction by reducing the concentration of H_2_O_2_ and thereby lowering the inhibitory effect of H_2_O_2_. Moreover, the reaction of H_2_O_2_ and SP produces CO_2_ and acetate that prevent the pH from drastically decreasing by establishing secondary buffering effects. The latter can be seen by the fact that the pH was around 6 at the end of the overnight reaction (Table 1). The higher activity of the enzyme in the presence of SP was also confirmed by the total consumption of glucose (Figures S7 and S8). When the glucose concentration was increased, the whole amount of SP was decarboxylated into acetate before the total consumption of the glucose in solution. Because of that, the H_2_O_2_ concentration increased in the reaction mixture, causing the protein to become inactive and nonreacted glucose to be detectable in ^1^H NMR spectra (Figures S7 and S8).

### Polymerization Reaction in Conditions of the Reduced Oxygen Level

To increase the yield of the reaction in a 96-well plate, the polymerization of 500 mM NAM with 5 mg/mL GOx was repeated under conditions of the reduced oxygen concentration, established by covering the reaction mixture with a layer of mineral oil without prior degassing (Table 1). Mineral oil eliminates the headspace on top of the reaction mixture and limits the diffusion of oxygen into the reaction media,^80,81^ thus reducing H_2_O_2_ production and accumulation, as well as media acidification. The reaction proceeded better than the reactions that were fully open to the air, resulting in a maximum yield of around 50% when no H_2_O_2_ scavengers were added. Low pH and a fully oxidized enzyme were observed at the end of the reaction (Table 1 and Figure S9), indicating a deactivation mechanism similar to the one involved in the open-air reaction. The yield greatly improved when catalase was added to the mixture, resulting in 84% monomer conversion. When SP was used as a H_2_O_2_ scavenger, yields above 85% were obtained at any glucose concentration, with a slightly lower yield when higher concentrations of SP were used, probably due to the reducing properties of SP that might quench the radical on the growing chain.

The final product of the reaction without SP or with 200 mM SP was analyzed in gel permeation chromatography (GPC), resulting in a *Đ* of 2.7 and a *M*_n_ of 1.7 × 10^5^ g/mol when SP was omitted and a *Đ* of 2.5 and *M*_n_ of 3.7 × 10^5^ g/mol when SP was used.

Moreover, the protein’s oxidation state was monitored for the first 14 h of the reaction by measuring the solution’s absorbance at 450 nm, where FAD exhibits a peak of absorbance that is absent in the FADH_2_ form (Figure S10). During the reaction in the lower oxygen regime, the reduced cofactor of GOx progressively oxidized back to FAD in the absence of catalase or SP. With catalase as a scavenger for H_2_O_2_, the oxidation of the cofactor was partially suppressed in the first 6 h, after which it proceeded at a similar rate than the cofactor oxidation in the absence of catalase or SP. This might be due to the progressive acidification of the media that can inactivate both GOx and catalase (the latter showing a decline in activity below pH 4 ^82^). The inactivation of catalase also increases the H_2_O_2_ concentration, thus contributing further to the inhibition of GOx. Importantly, no oxidation was observed when SP was used, which demonstrates its good capability to remove H_2_O_2_ from the solution and maintain a constant pH.

GOx can thus deoxygenate the reaction mixture and keep the oxygen levels of the solution low enough for the polymerization to proceed, as long as the reoxygenation of the solution from the headspace is stopped by covering the reaction mixture with mineral oil and if SP is used to eliminate the H_2_O_2_ that is produced in the deoxygenation reaction and to stabilize the pH. Under these conditions, GOx can fulfill the dual role of removing oxygen and, at the same time, initiate the polymerization reaction under mild conditions.

### Fluorescent Assay to Follow the Polymerization in a 96-Well Plate

Given the possibility of performing the polymerization in 96-well plates without the need for degassing, thus making the procedure easier and screenable, the possibility of following the polymerization reaction in real-time, without the need for NMR analysis, became interesting. To achieve this, the lipophilic dye Nile Red was selected to create a suitable assay for detecting polymer formation. The dye is known to stain many polymers and microplastics by switching on its fluorescence after interacting with hydrophobic environments,^83^ thus revealing the presence of the polymers. The polymerization of a 500 mM NIPAm solution with 5 mg/mL GOx, 200 mM glucose, and 0.2 mg/mL catalase or 200 mM SP as H_2_O_2_ scavenger was performed, using mineral oil to create a low-oxygen level regime. Furthermore, a series of controls lacking one or more of the essential components of the reaction, i.e., monomer, glucose, and GOx, were included. The reactions were run overnight at 30 °C to avoid PNIPAm precipitation, with 10 μg/mL Nile Red per well (Figure 3a). The polymerization was run in the dark for 18 h, and Nile Red emission was then collected at 650 nm upon excitation at 550 nm (Figure S11). A strong increase in fluorescence was observed for all conditions that resulted in polymer formation. The control reactions showed no increase in Nile Red fluorescence, confirming that the dye stained the polymer and that Nile Red did not interact with any other components of the reaction.

**Figure 3:**
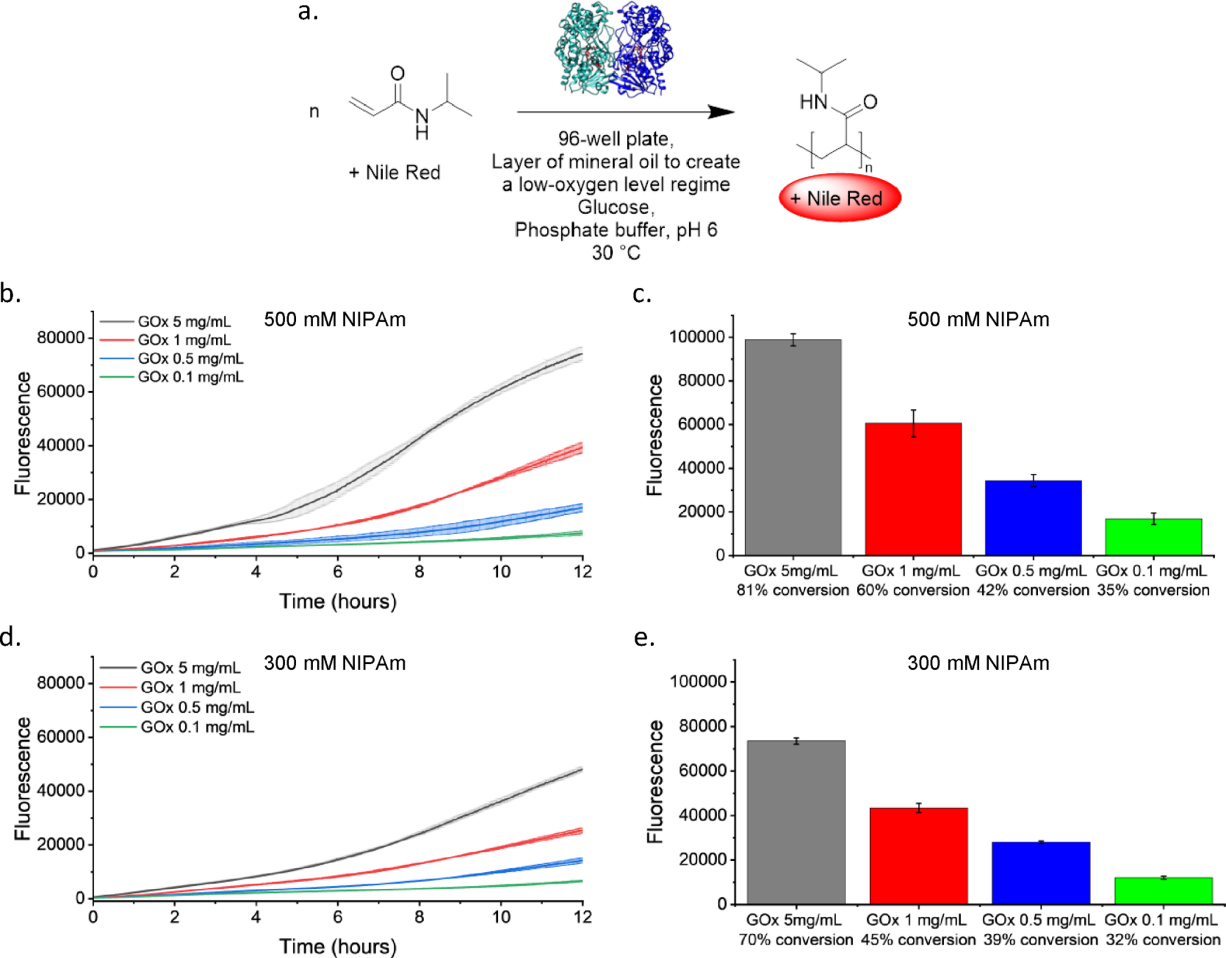
Nile Red fluorescent assay to follow the GOx-initiated polymerization of NIPAm in 96-well plates. (a) Schematic representation of the reaction condition. (b) Nile Red assay at a NIPAm concentration of 500 mM. The assays were performed at different concentrations of GOx (0.1, 0.5, 1, and 5 mg/mL), with 200 mM glucose and 200 mM SP as a hydrogen peroxide scavenger at 30 °C in a low-oxygen regime. (c) Fluorescence emission of the assays shown in panel (b) after 18 h of reaction time. The conversion, as determined by ^1^H NMR spectroscopy, is also indicated. (d) Nile Red assay at a NIPAm concentration of 300 mM. The assays were performed at different concentrations of GOx (0.1, 0.5, 1, and 5 mg/mL) with 200 mM glucose and 200 mM SP as a hydrogen peroxide scavenger at 30 °C in a low-oxygen regime. (e) Fluorescence emission of the assays shown in panel (d) after 18 h of reaction time. The conversion, as determined by ^1^H NMR spectroscopy, is also indicated. For each measurement, the mean ± SD is reported; *n* = 3.

To correlate the fluorescence intensity of Nile Red to the conversion measured by NMR spectroscopy and the GOx concentration, reactions at different concentrations of GOx were repeated in the presence of 200 mM SP and 300 or 500 mM NIPAm (Figure 3b–e, Figure S12, and Table S4). Nile Red fluorescence increased over time with conversion and increased more slowly at a lower enzyme concentration. Moreover, the conversion and fluorescence intensity at 18 h of reaction were proportional to the amount of GOx used (Figure S12). Thus, Nile Red fluorescence correlates with the catalyst concentration and conversion, and it can be exploited to follow the polymerization in a 96-well format, e.g., to screen for good reaction conditions.

### Mechanistic Investigation of the Reaction

The formation of radicals during the reaction of 500 mM NAM with 5 mg/mL GOx and 200 mM glucose was detected by using dihydrorhodamine 123 (DHR). This compound is a profluorescent dihydroderivative of the fluorescent dye rhodamine 123. It is most commonly used for the detection of reactive oxygen species in cells.^84^ DHR can be used to detect a variety of radicals, so we hypothesized that it might be useful for the detection of monomer radicals’ formation. The assay was performed in the presence of 400 mM SP to scavenge any H_2_O_2_ produced. When NAM was added to the reaction mixture in a low-oxygen regime, an increase in fluorescence could be detected, (Figure S13), indicating that radicals formed.

To get a better understanding of the novel activity of GOx on the molecular scale, the interaction between two monomers (NAM or NIPAm) and GOx was studied by docking simulations. Both monomers showed interactions with the reduced FAD cofactor and the active pocket of the enzyme, with a dissociation constant (*K*_d_) in the micromolar range (Table S4), as calculated by the YASARA Structure. The monomers docked in the active pocket of the enzyme showed the oxygen atom of the acrylamide group closest to the nitrogen N5 of the FAD cofactor (which could donate a proton and an electron) with a distance below 2.2 Å (Table S5 and Figure 4). Moreover, the analysis of the docking revealed a hydrogen bond formed between the two atoms (Figure 4). This observation suggests the oxygen of the monomer as an electron acceptor moiety that could be reduced by the flavin cofactor, leading to the formation of radical species from the monomer and the subsequent start of the polymerization.

**Figure 4:**
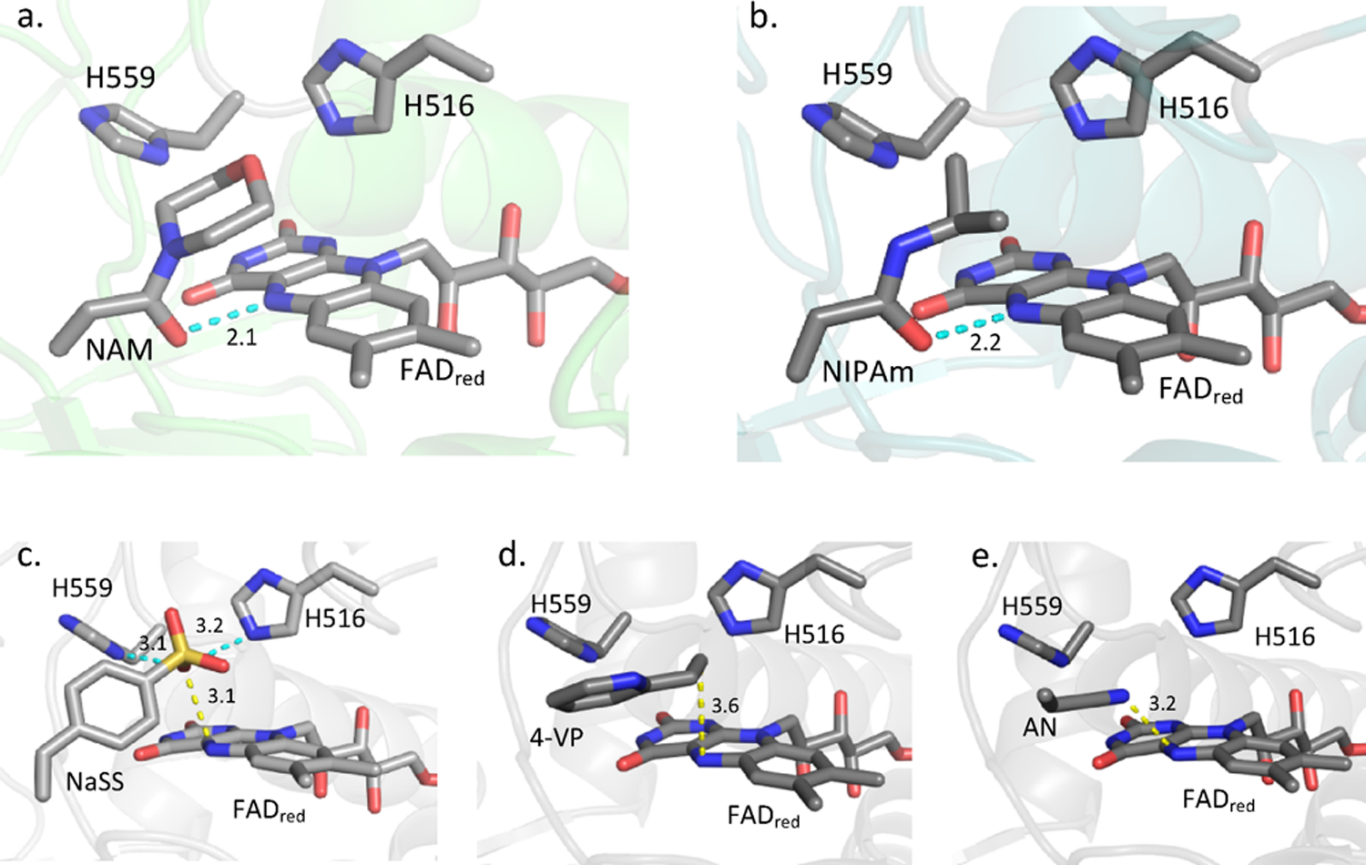
Models of the active site of GOx after rigid molecular docking of various monomers. (a) NAM. (b) NIPAm. (c) NaSS. (d) 4VP. (e) AN. Cyan dotted lines indicate hydrogen bonds, while yellow dotted lines indicate distances. Dockings were performed by the YASARA structure and analyzed by PyMOL.

To test if the enzyme was also active toward monomers with a different structure than those derived from acrylamide and methacrylate investigated above, three more water-soluble monomers, namely, acrylonitrile (AN), 4-vinylpyridine (4VP), and sodium styrenesulfonate (NaSS), were tested. The docking of those monomers showed *K*_d_ values in the micromolar range for 4VP, the millimolar range for AN, and the lower micromolar range for NaSS. Again, when oxygen atoms were present in the molecular structure of the monomer (i.e., NaSS), the docking showed an orientation of the substrate such that oxygen atoms were oriented toward the reduced cofactor and hydrogen bonds were forming between the substrate and the key His residues of the enzyme (Figure 4 and Table S5).

These new monomers were then tested for polymerization. NaSS was successfully polymerized by GOx under anoxic conditions (Figure S14). In contrast, the overnight reactions of 4VP and AN produced no polymer. AN was tested at concentrations between 500 and 700 mM, but no polymer was found in any condition tested (Figure S14). The low *K*_d_ obtained from the docking of AN in the GOx active site might explain the result. 4VP formed a bright red byproduct, and the pH of this reaction mixture increased to 8.5 after 18 h of reaction in anoxic conditions, but no polymer was found in any of the reactions, which were carried out at least twice. These results further suggest a direct involvement of oxygen atoms in the molecular structure of the monomers for the formation of radical species that could then start the reaction.

Our findings allow us to propose a possible mechanism for the GOx-initiated polymerizations (Figure 5). The cofactor of the enzyme is reduced by glucose to form FADH_2_ (or FADH^-^ at pH 6). Then, the cofactor transfers a single electron to a monomer, likely through the oxygen atom of the carbonyl bond, followed by proton transfer to the monomer. It could also be first a protonation and then an electron transfer to the monomer, or a concerted electron and proton transfer, but these sequences of events would be less aligned with the known mechanism of GOx with O_2_ as a substrate.^2^ In any case, electron transfer, coupled with proton transfer, might occur again for a second monomer, allowing the cofactor to return to its FAD form. As a result, the monomers are reduced by the enzyme to form free radical species that start the polymerization reaction.

**Figure 5:**
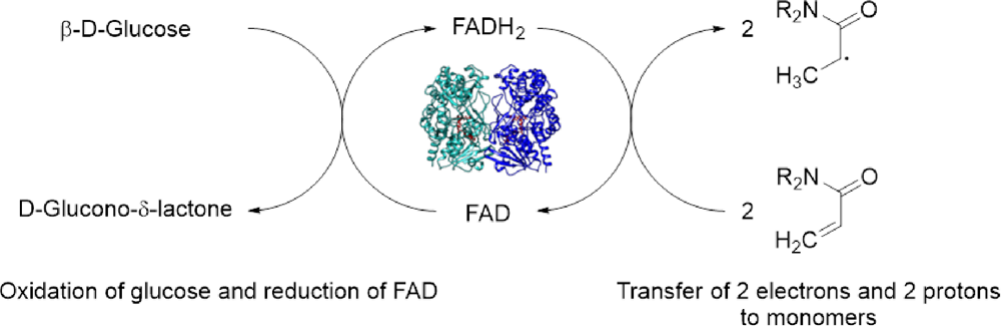
Proposed mechanism for the GOx-catalyzed polymerization. The cofactor of the enzyme is reduced by glucose in solution to form FADH_2_. The oxidation of the cofactor to FAD can reduce the monomer molecules to form free radical species able to start the polymerization reaction.

### GOx Stability and Activity

The stability of glucose oxidase was tested in the presence of different monomers by measuring the fluorescence of its cofactor. FAD emits light with an emission maximum at 540 nm upon excitation at 450 nm only when it is free in solution. The release of the cofactor from the protein can, therefore, be monitored and used to determine the protein’s stability under different conditions. The enzyme was incubated for 30 min at 37 °C with 500 mM of various monomers, and no significant change in GOx stability was observed under any of the tested conditions (Figure 6a). When the protein was incubated for a longer time (18 h at 37 °C) with 500 mM of various monomers, the fluorescence of FAD increased, indicating a partial unfolding with consequent release of the cofactor in the solution for many monomers tested, with the worst being 4VP, AAm, and HEMA (Figure 6b). In contrast, the enzyme remained stable in the presence of NaSS.

**Figure 6:**
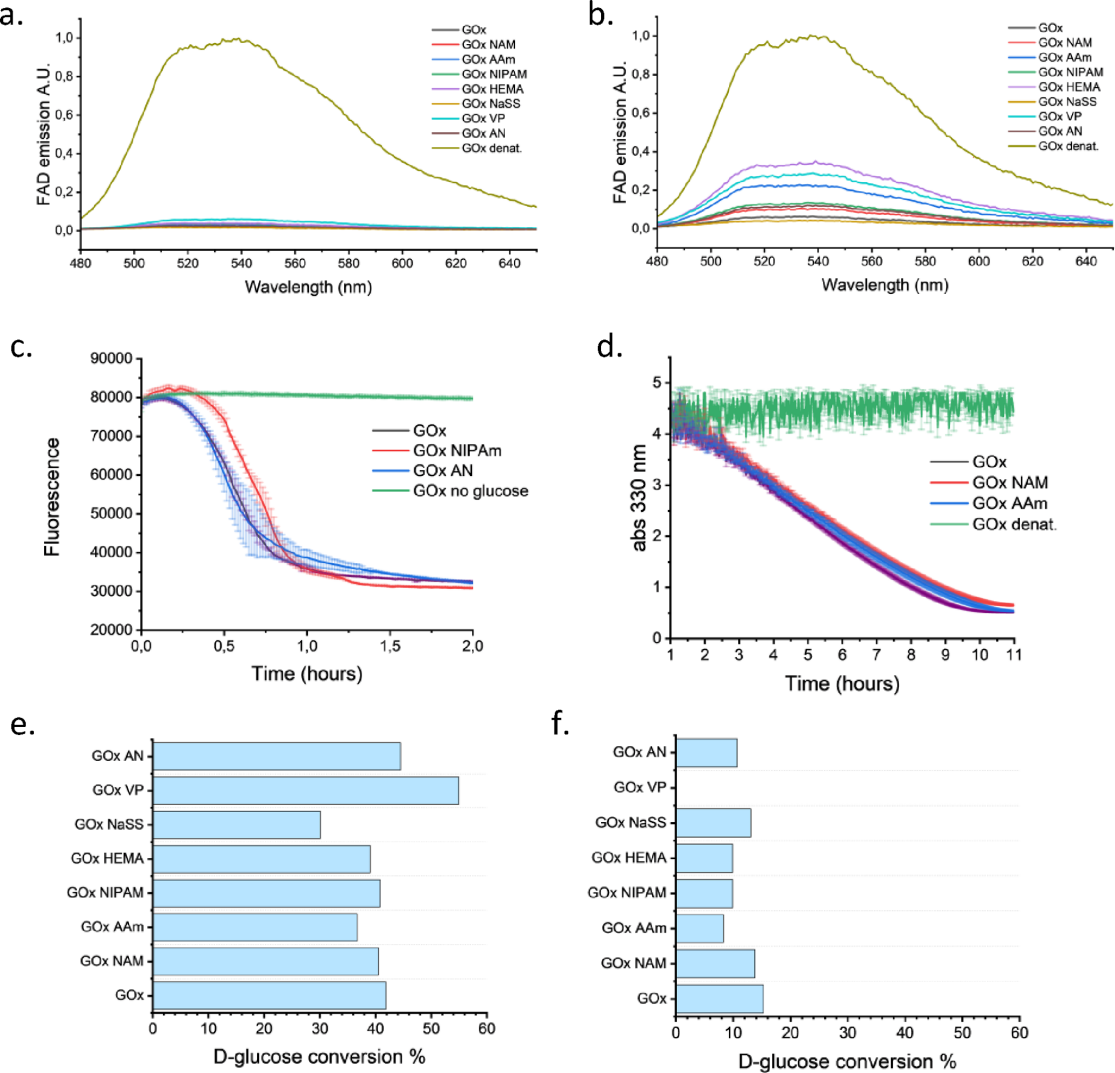
Stability and activity of GOx in the presence of various monomers. (a, b) GOx stability measured by the fluorescence of its cofactor FAD, which only fluoresces when it is free in solution. Fluorescence spectra of GOx solutions after incubation of GOx with various monomers at 500 mM (a) for 30 min at 37 °C and (b) for 18 h at 37 °C. (c) Glucose oxidation activity of GOx in the presence of NIPAm or AN measured by the decrease of fluorescein’s fluorescence emission upon reduction of the pH due to the production of gluconic acid from oxidation of the glucose. 500 mM of monomers were added to 5 mg/mL GOx at 37 °C; fluorescein concentration in well: 1 mM; excitation: 488 nm; emission: 520 nm. For each measurement, the mean ± SD is reported; *n* = 3. (d) Glucose oxidation activity of GOx in the presence of NAM or AAm followed by measuring the consumption of SP upon reaction with H_2_O_2_ through SP’s absorbance peak at 330 nm. 500 mM monomers were added to 5 mg/mL GOx at 37 °C in wells open to air. For each measurement, the mean ± SD is reported; *n* = 3. (e, f) Glucose oxidation activity of GOx in the presence of various monomers measured by the conversion of glucose to gluconic acid through ^1^H NMR spectroscopy. Conversion of glucose in the presence of the 500 mM monomer (e) and after incubating the enzyme for 18 h with the monomer prior to the activity test (f). For the first set of experiments, 5 mg/mL GOx was allowed to react with 200 mM glucose in open air, in the presence of the 500 mM monomer at 37 °C for 1 h. For the latter set of experiments, 5 mg/mL GOx was incubated with the 500 mM monomer at 37 °C for 18 h. After that, 200 mM glucose was added to the mixture, which was then allowed to react at 37 °C for 1 h. Three reactions were prepared for each condition and combined for NMR analysis.

Next, the native glucose oxidation activity of the protein was tested in the presence of different monomers in fully open-air conditions, when no polymerization happens or just low monomer conversion can be achieved. To this end, fluorescein’s pH-dependent fluorescence, SP consumption, and glucose-to-gluconic acid conversion were employed. Catalytic assays based on the usual enzymatic cascade of GOx and horseradish peroxidase to study GOx activity were not used to avoid potential errors due to a possible loss of activity of the second enzyme under unfavorable conditions.

Fluorescein is a pH-sensitive dye with low fluorescence at low pH and can therefore be used as a pH indicator to determine GOx activity. Following the oxidation of glucose, gluconic acid is formed in the solution, bringing the pH to values below 2. With this assay, no remarkable differences were found in the GOx activity in the presence of 500 mM NIPAm or AN, indicating that the enzyme’s activity is not influenced by these monomers (Figure 6c). SP consumption was followed by monitoring the decrease of the absorbance peak at 330 nm due to decarboxylation of SP into acetate. The presence of 500 mM NAM or AAm did not change the SP consumption, proving the consistency of GOx activity (Figure 6d). Finally, the GOx-catalyzed glucose-to-gluconic acid conversion in conditions fully open to air, without hydrogen peroxide scavengers, was followed by NMR spectroscopy in the presence of 500 mM NAM, AAm, NIPAm, HEMA, NaSS, 4VP, or AN. GOx retained similar activity across all conditions tested; only NaSS led to a slightly lower conversion of glucose compared to the enzyme alone (30 vs 40% conversion) (Figure 6e and Figure S15). Thus, the monomers did not inhibit GOx’s glucose oxidation activity, which is in line with the results discussed above. Interestingly, the presence of 4VP increased the enzyme’s activity, resulting in glucose consumption exceeding 50%. The final pH of the solution might explain this. In the presence of 4VP, the pH does not drop during the reaction but increases slightly to 8.5, likely keeping the enzyme in a more favorable pH range for a longer period.

The same experiment was then repeated, but the enzyme activity was measured after an overnight incubation with the monomers at 37 °C, before the addition of glucose to the mixture. Also in this case, GOx exhibited the same activity in the presence or absence of monomers, except for 4VP, which completely deactivated GOx, resulting in no gluconic acid production (Figure 6f and Figure S16).

Overall, none of the monomers significantly affected the enzyme’s stability and activity in the first hours of the experiments, and GOx retained its glucose oxidation activity under a wide range of conditions. Only prolonged exposure at 37 °C to certain monomers, such as 4VP, had a negative effect on GOx stability and activity.

Finally, the stability of the enzyme was tested during the polymerization reaction in a reduced oxygen concentration regime using NAM as a model monomer. To follow the folding state of GOx, the dye Sypro orange (which increases its fluorescence when bound to hydrophobic patches of the protein) was used, and the results were compared to those obtained from FAD emission experiments (Figure S17).

Overall, the polymer formation did not result in any significant unfolding of the protein compared to the condition without the monomer, proving the compatibility of the polymerization with the enzyme. The addition of SP to the reaction mixture provided extra protection to the enzyme, resulting in a lower release of FAD during the 15 h analyzed and, thus, better protein stability, probably due to the stabilization of the pH and elimination of H_2_O_2_.

## Conclusions

In this study, we reveal GOx from *A. niger* as a catalyst for the creation of radical species from vinyl monomers, thereby initiating free radical polymerization without the need of additional initiators or the irradiation with light. This represents a previously unknown catalytic activity of this promiscuous enzyme. Unlike conventional systems that require external initiators or light, GOx promotes polymerization through direct electron and proton transfer to the monomer in anoxic or low-oxygen conditions, producing polymers of high molecular weight in less than 24 h. Our results confirm the successful polymerization of various monomers, achieving conversion above 90% under mild reaction conditions. Thanks to GOx’s intrinsic ability to remove dissolved oxygen, the reaction could even be carried out without degassing the solutions, and it could be scaled down to a 96-well plate format. A polymerization screening based on Nile Red was also developed to follow the polymerization in multiwell plates, which opens the possibility for high-throughput screening of reaction conditions or of engineered variants of GOx. Furthermore, the addition of sodium pyruvate as a hydrogen peroxide scavenger significantly improved the reaction efficiency in a low-oxygen regime. Various monomers were successfully docked in the active site of the flavoprotein, with an orientation of these small molecules that supports the possibility of a direct electron transfer between the enzyme and the monomers.

Polymers were easily synthesized under mild, aqueous, and semiopen-air conditions, offering a biocatalytic alternative to producing polymers without the need for transition metal complex catalysts, conventional initiators, or irradiation with light. This might make the polymerization process greener and more sustainable, and importantly, it allows the integration of simple enzyme-mediated radical polymerizations into synthetic biology systems and living cells (e.g., for cell-surface engineering with synthetic polymers). Moreover, by addition of a RAFT agent, the polymerization could be turned into a controlled radical polymerization. Finally, our results demonstrate that when GOx is used in any kind of polymerization setting, whether these are controlled radical polymerizations, deoxygenations, enzymatic cascade reactions, or light-induced polymerizations, one should take into account that some of the polymerization activity could arise from the direct electron and proton transfer from the reduced enzyme to the monomers.

## Experimental Section

### Materials

Monomers, Nile Red, fluorescein sodium salt, glucose, sodium pyruvate, heavy mineral oil (0.862 g/mL), and HEBIB were purchased from Sigma-Aldrich at the highest purity available and used directly without further purification unless otherwise stated. Glucose oxidase from *A. niger* type X-S (lyophilized powder, 100,000–250,000 units/g solid) and catalase from bovine liver were purchased from Sigma-Aldrich. Buffers were prepared in loco. Dihydrorhodamine 123 dihydrochloride was purchased from Biotium. Sypro orange was purchased from Thermo Fisher Scientific.

### Methods

^1^H NMR spectra were recorded on a Bruker AVA300 spectrometer (300 MHz) at 298 K in deuterated solvents. For GPC measurements, samples were passed through a column of alumina oxide to remove the enzyme and then dried completely under reduced pressure by using a Biotage V-10 Touch evaporation system. The samples were then redissolved in DMF (+ 1 mg/mL LiBr) by shaking for several hours before being filtered through a 0.45 μm PTFE syringe filter. The measurements were performed on a PSS/Agilent 1260 Infinity instrument equipped with a refractive index (RI) detector. Two PSS Gram Linear columns were used at 50 °C. Samples were run at a flow rate of 1 mL/min. PSS ReadyCal-kit PEO/PEG was used as a reference. The number of average molar mass (*M*_n_) and dispersity (*Đ*) values were determined with the PSS WinGPC software. A BMG Labtech CLARIOstar Microplate Reader was used for absorbance and fluorescence assays. Fluorescent experiments in 96-well plates were carried out with bottom optic setting in dark-clear bottom NUNC 96-well plates with a nontreated surface, while other types of reactions in 96-well plates were carried out in transparent 96-well F bottom plates covered with a lid and aluminum foil.

### General Polymerization Procedure in Aqueous Solution

Liquid monomers containing inhibitors were passed through a plug of basic alumina to remove the inhibitors, while solid monomers were used as is. The monomer, glucose, and pyruvate, when present, were mixed in 50 mM PB, pH 6 and bubbled with argon for 1 h at room temperature. Commercial glucose oxidase from *A. niger* type X-S was bubbled in solution with argon for the initial screenings, while it was kept under a constant flux of argon in a powder form in a closed Schlenk flask (10 mL) for 1 h at room temperature for the optimized reactions. The reaction was started by transferring the mix of monomer, glucose, and sodium pyruvate into the Schlenk flask through a degassed syringe, and the reaction mixture was let to react in a water bath at 37 °C closed with aluminum foil in a dark room under a constant argon atmosphere.

GOx (1 mg/mL), 200 mM glucose, 200 mM sodium pyruvate, and 300 mM monomer were used for the initial screenings, and 5 mg/mL GOx, 200 mM glucose, 200 mM sodium pyruvate, and 500 mM monomer were used for the optimized reaction in anoxic conditions. When H_2_O_2_ was used, a commercial 35% H_2_O_2_ stock solution, a monomer solution (500 mM) in 50 mM PB pH 6, with or without 200 mM pyruvate, and 200 mM glucose or 5 mg/mL GOx were degassed separately under argon for 1 h. After that time, a quantity of degassed 35% H_2_O_2_ solution was transferred under an inert atmosphere to the reaction mix to achieve a final H_2_O_2_ concentration of 1 M. For the reactions where the reaction mix was sampled at various time points: 200 μL samples were taken at different time points through a degassed syringe and quickly mixed with 100 μL of a freshly made solution of 200 mM hydroquinone in D_2_O. NMR samples were prepared by mixing the samples in 400 μL of DMSO-*d*_6_.

Monomer conversion was calculated from ^1^H NMR spectra (200 μL samples +450 μL of deuterated solvent) by integrating the peak derived from one of the protons of the double bond of the monomer (region around 6 ppm) and integrating the same proton in the backbone of the polymer (around 2 ppm). The following formula was then applied:

$$\mathrm{conversion}=\left(\frac{\frac{\ln(p)}{H(p)}}{\left(\frac{\ln(p)}{H(p)}\right)+\left(\frac{\ln(m)}{H(m)}\right)}\right)\cdot 100$$

where ln­(p) is the integration of the polymer peak corresponding to the backbone proton, *H*(p) is the number of protons contributing to the polymer peak considered, ln­(m) is the integration of the monomer peak corresponding to one proton of the double bond, and *H*(m) is the number of protons corresponding to the monomer peak considered (i.e., 1).

### Nile Red Polymerization Assay

A Nile Red stock solution (1 mg/mL in DMSO) was prepared and stored at 4 °C. For the assay, a final concentration of 10 μg/mL was used in a final volume of 200 μL. The reaction mixture was prepared in a 96-well dark plate with clear unfunctionalized bottom by thoroughly mixing glucose, hydrogen peroxide scavenger (when present), and Nile Red before adding GOx. The well was then filled to the top with heavy mineral oil, and the monomer was added to reach a final reaction volume of 200 mL. The reaction was then carried out in the dark of the measuring compartment of the plate reader at 30 °C under constant shaking. Wells were only irradiated briefly (every 70 s) for analytical purposes. Nile Red was excited at 550 nm, and the fluorescence emission was recorded at 630 nm.

### DHR Assay

A dihydrorhodamine 123 dihydrochloride stock solution was prepared in DMSO (10 mg/mL) and used immediately or kept at −20 °C for no longer than 2 weeks. A final concentration of 387 μM was used for the assay in a final volume of 100 μL. The reaction mixture was prepared as described for the Nile Red assay. The reaction was carried out in the dark of the measuring compartment of the plate reader at 37 °C under constant shaking. Wells were only irradiated briefly (every 90 s) for analytical purposes. DHR was excited at 487 nm, and the fluorescence emission was recorded at 535 nm.

### Docking Simulations

Rigid dockings were performed by the YASARA structure using an AUTODOCK Vina algorithm, 100 runs, pH 6, with a simulation box of 15 Å centered on the N5 atom of FAD. The crystallography structure of GOx with PDB entry 1cf3 was used for the analysis and the dimeric structure reconstructed by homology with the crystallography structure of GOx from *Penicillium amagasakiense* (PDB entry 1gpe). The reduced form of FAD was used, with both catalytic histidines protonated and the cofactor holding a negative charge on N1 and a proton on N5, following energy minimization in water. The results of the docking were analyzed in PyMOL

### Fluorescein pH Assay

Fluorescein sodium salt stock solution was prepared in DMSO (100 mM), and a final concentration of 1 mM was used in the well. The reaction of 5 mg/mL GOx, 200 mM glucose, and 500 mM monomer in 50 mM PB pH 6 was performed in open air at 37 °C in a 96-well plate closed with a microplate lid to avoid evaporation. The reactions were carried out in the darkness of the measuring compartment of the plate reader, and wells were only irradiated briefly (every 60 s) for analytical purposes. Fluorescein was excited at 480 nm, and the fluorescence emission was recorded at 530 nm.

### Sodium Pyruvate Consumption Assay

SP was used at a final concentration of 270 mM, with 5 mg/mL GOx, 680 mM glucose, and 500 mM monomer in 50 mM PB at pH 6. The reaction was performed in the open air at 37 °C in the plate reader under constant shaking. The reactions were carried out in the darkness of the measuring compartment of the plate reader, and wells were only irradiated briefly (every 90 s) for analytical purposes. The absorbance at 330 nm was measured every 90 s.

### Glucose Conversion to Gluconic Acid

GOx (5 mg/mL) was mixed with 500 mM monomer and 200 mM glucose in a final volume of 200 μL of PB pH 6 and incubated overnight at 37 °C under shaking. The 96-well plate was covered with a lid (with condensation rings to avoid evaporation) and aluminum foil. Triplicates were prepared on the same plate. Reaction mixture (200 μL) was then mixed with 400 μL of D_2_O and analyzed by ^1^H NMR spectroscopy. The percentage conversion of glucose was calculated from the spectra.

## Supplementary Material



## Notes

GPC data and monomer conversion, NMR spectra, fluorescent assays curves, photos of reaction mixtures and experimental setup, SEC elugram, UV–vis spectra, cofactor reoxidation measurements, and docking results (PDF)
