# Supplementary material for: Glucose Oxidase Initiates Radical Polymerizations by Direct Electron Transfer to Monomers
Source: Biomacromolecules. 2025 Sep 25;26(10):7038–50. doi: 10.1021/acs.biomac.5c01372 (PMC12522130; doi:10.1021/acs.biomac.5c01372)
Supplement: Supplementary file 1 [file bm5c01372_si_001.pdf]

SUPPORTING INFORMATION

Glucose oxidase initiates radical polymerisations by direct electron transfer to monomers

Eleonora Ornati,[1,2] Iuliia Ushakova,[1] Nico Bruns[1,2,*]

[1]Department of Chemistry and Centre for Synthetic Biology, Technical University of Darmstadt, Peter-Grünberg-Str. 4, 64287 Darmstadt, Germany

[2]Department of Pure and Applied Chemistry, University of Strathclyde, Thomas Graham House, 295 Cathedral Street, Glasgow G1 1XL, U.K.

*nico.bruns@tu-darmstadt.de

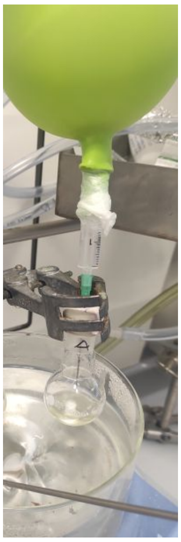

Figure S1. Photo of an anoxic polymerization setup after 18 h of reaction at 37°C using reduced GOx as the catalyst. After 1 hour of thoroughly degassing with argon, the reaction mixture was kept under argon atmosphere with the use of a balloon filled with the inert gas argon.

Table S1. Polymerisation of 300 mM NIPAm under anoxic conditions that proceeded without an initiator.

Monomer	Glucose	GOx	Initiator	Conversion
NIPAm	Yes	Yes	Yes	14%
	Yes	Yes	No	26%
	No	Yes	Yes	-
	Yes	No	Yes	-

For the reaction, 1 mg/mL GOx, 200 mM glucose and 15 mM HEBIB were used after 1 hour of degassing with argon. Polymer formation was followed by ^{1}H NMR spectroscopy.

Table S2. Screening of the polymerisation of various monomers with the GOx system.

Monomer	Glucose	GOx	Polymer
NIPAm	Yes	Yes	Yes
	Yes	No	No
	No	Yes	No
NAM	Yes	Yes	Yes
	Yes	No	No
	No	Yes	No
AAm	Yes	Yes	Yes
	Yes	No	No
	No	Yes	No
HEMA	Yes	Yes	Yes
	Yes	No	No
	No	Yes	No
PEGMA	Yes	Yes	Yes
	Yes	No	No
	No	Yes	No

300 mM of the monomer was polymerised with 1 mg/mL GOx and 200 mM glucose under anoxic conditions at 37 °C overnight. Controls without protein or glucose led to no polymer formation. Polymer formation was followed by ^{1}H NMR spectroscopy.

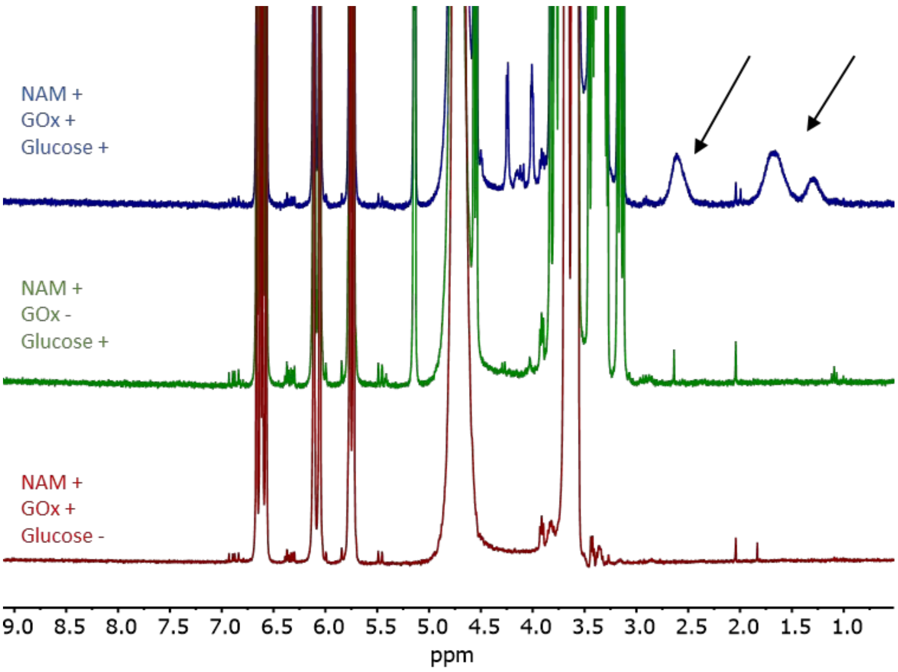

Figure S2. ^{1}H NMR spectra of GOx-initiated polymerisations of NAM. Polymer was obtained under anoxic conditions only when a reduced GOx was used, i.e. in the presence of glucose. Black arrows indicate the backbone peaks of PNAM.

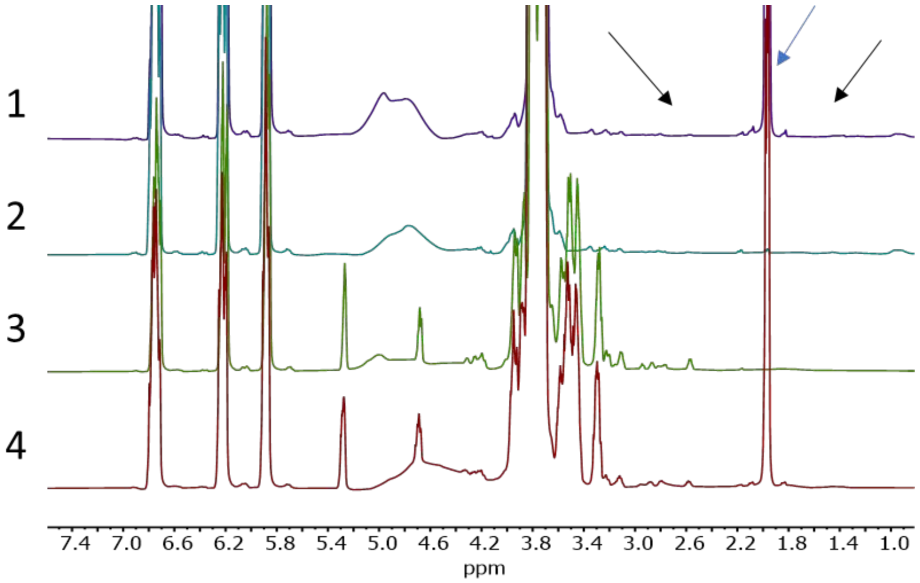

Figure S3. ^{1}H NMR spectra of control reactions to rule out that hydrogen peroxide initiates the polymerisations. The use of 1.5 mM H_2O_2 with 500 mM NAM and 5 mg/mL GOx (1 and 2) or 200 mM glucose (3 and 4) in anoxic conditions did not lead to the formation of polymer chains, as indicated by the absence of the typical backbone peaks (black arrows). For spectra 1 and 4: the addition of 200 mM SP to the reaction mixture led to the complete decarboxylation of SP into acetate, as indicated by the absence of the SP peak at 2.3 ppm and the presence of the acetate peak at 2 ppm (blue arrow).

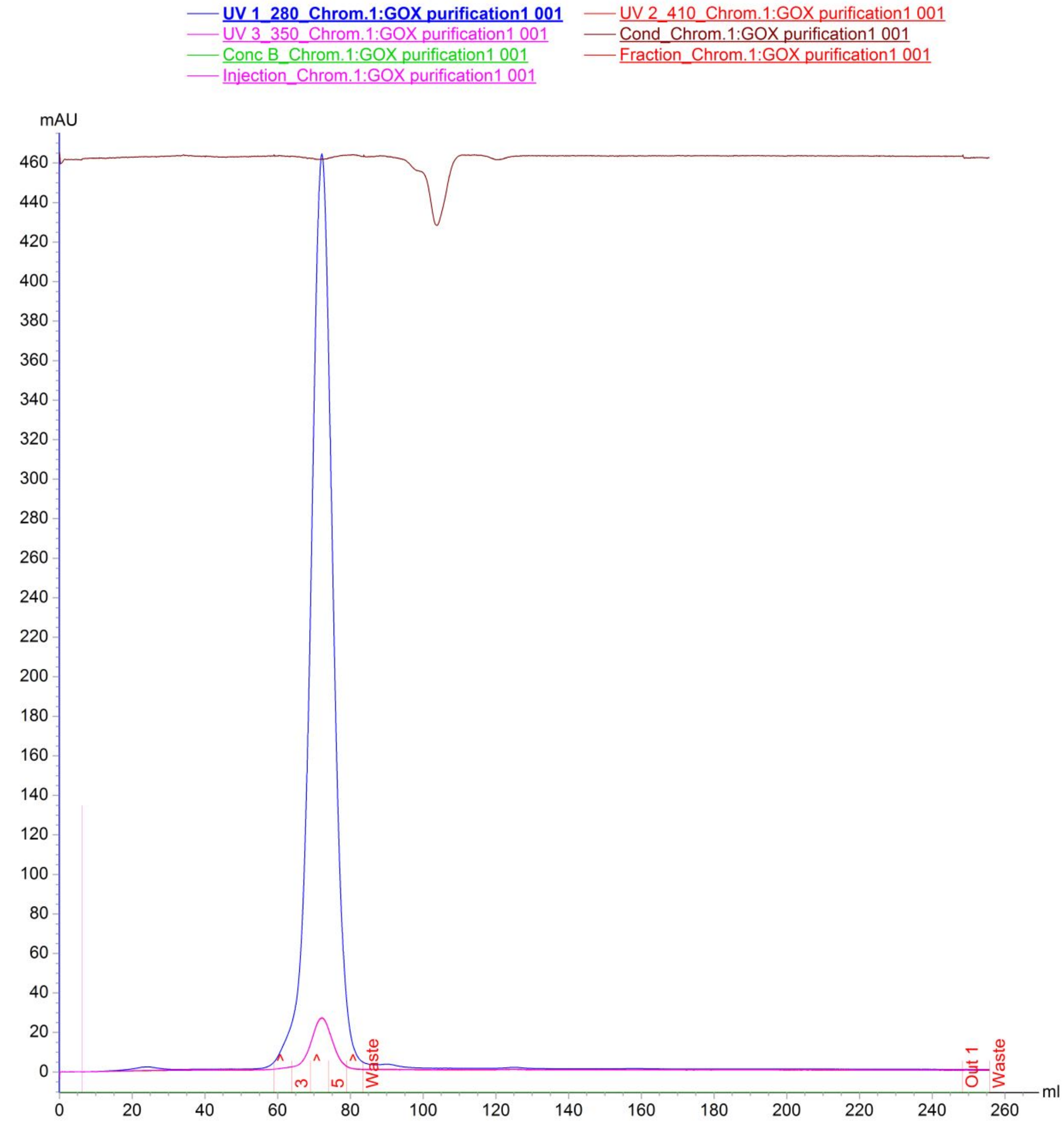

Figure S4. Elugram of the SEC run on AKTA system to purify the commercial GOx. The fractions 3 to 5 were collected and concentrated with a 10kDa centrifugal filter before being run on a PD-10 column to change the solvent and further purify the protein before being tested for the reaction.

Table S3. Monomer conversion, molecular weight (M_n) and polydispersity (Đ) at different time points of a typical GOx-initiated polymerisation of NAM.

	**Anoxic + SP**			**Anoxic - SP**		
Time (hour)	Conv. (%)	M_n (g mol^{-1})	Đ	Conv. (%)	M_n (g mol^{-1})	Đ
2	11.5	$4.98 \bullet 10^5$	2.07	10.7	$4.56 \bullet 10^5$	2.03
6	23.6	$3.72 \bullet 10^5$	2.51	30	$4.66 \bullet 10^5$	2.01
22	92	$2.87 \bullet 10^5$	2.8	93	$4.59 \bullet 10^5$	2.4

For the reaction, 500 mM NAM was reacted with 5 mg/mL GOx and 200 mM glucose in anoxic conditions, with (+ SP) and without (- SP) 200 mM SP.

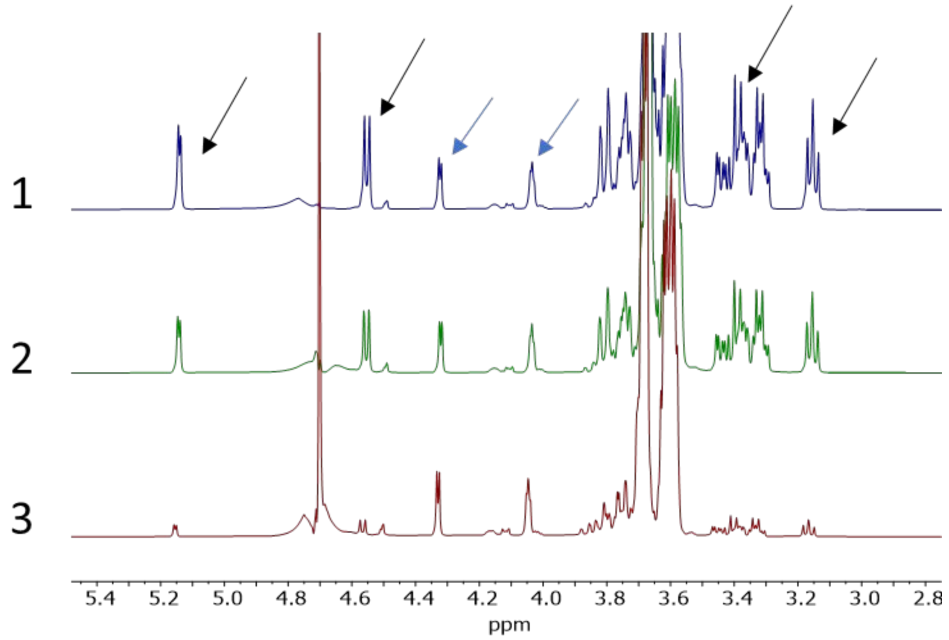

Figure S5. ^{1}H-NMR spectra of open-air reactions of GOx and NAM in the presence of different concentrations of glucose, without any H_2O_2 scavenger. 1) Glucose 1 M – monomer conversion <1%; 2) Glucose 500 mM – monomer conversion <1%; 3) Glucose 200 mM – monomer conversion <1%. Black arrows indicate unique glucose peaks, while blue arrows indicate unique peaks of gluconic acid. In all conditions tested, glucose was still present at the end of the reaction, indicating that the protein was deactivated before glucose was completely consumed.

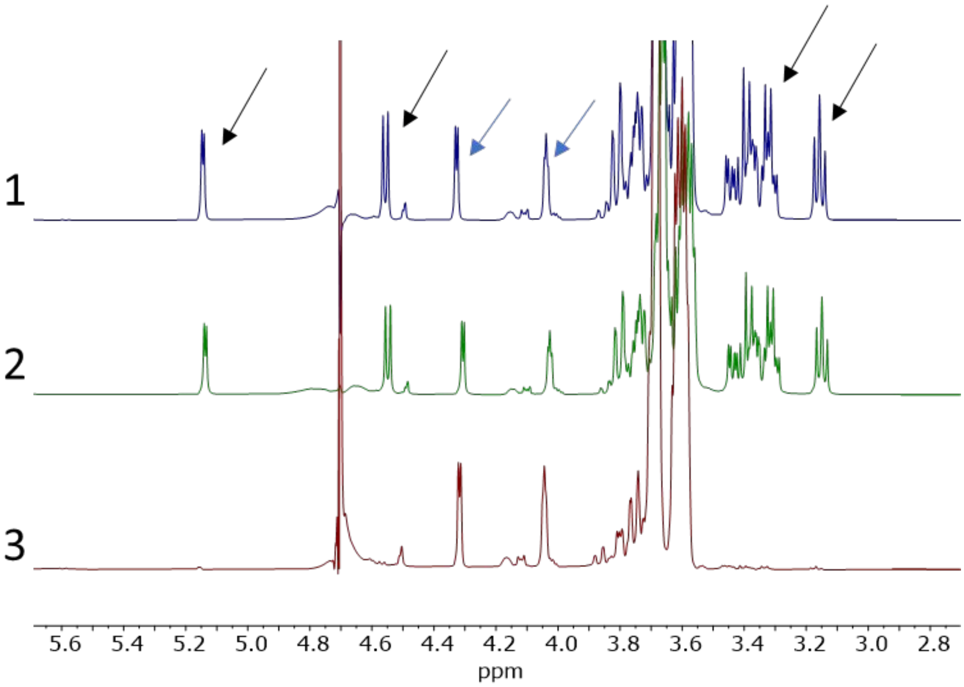

Figure S6. ^{1}H-NMR spectra of open-air reactions of GOx and NAM in the presence of different concentrations of glucose, with 0.2 mg/mL catalase as H_2O_2 scavenger. 1) Glucose 1 M – monomer conversion 7%; 2) Glucose 500 mM – monomer conversion 7%; 3) Glucose 200 mM – monomer conversion 1%. Black arrows indicate unique glucose peaks, while blue arrows indicate unique peaks of gluconic acid. Total glucose consumption was achieved when a low concentration of the sugar was used, indicating a prolonged activity of GOx compared to the condition without H_2O_2 scavengers (Figure S3), likely due to the removal of H_2O_2 by the catalase.

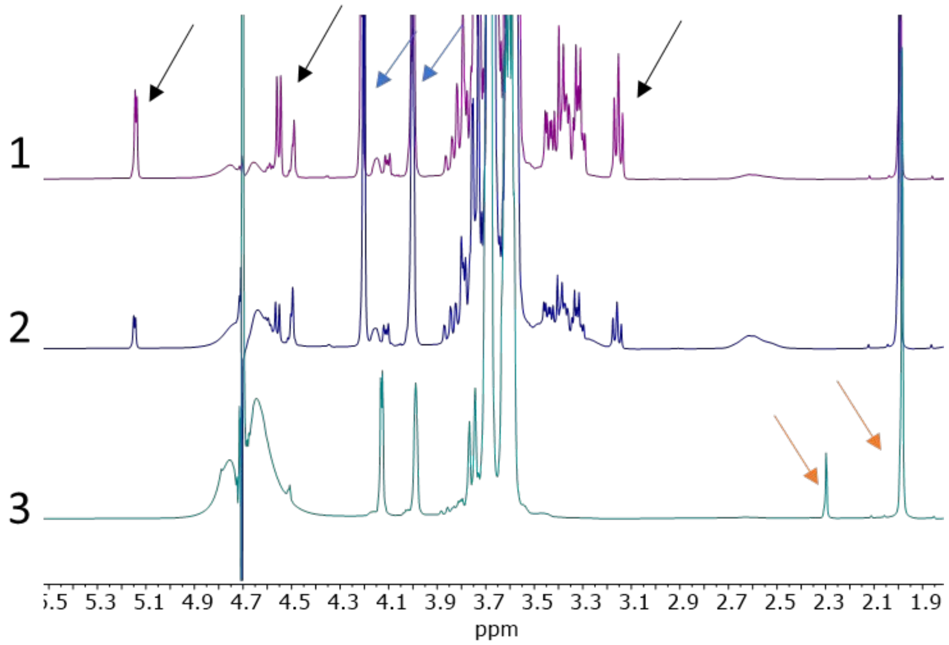

Figure S7. ^{1}H-NMR spectra of open-air reactions of GOx and NAM in the presence of different concentrations of glucose, with 200 mM SP as H_2O_2 scavenger. 1) Glucose 1 M – monomer conversion 33%; 2) Glucose 500 mM – monomer conversion 31%; 3) Glucose 200 mM – monomer conversion 20%. Black arrows indicate unique peaks of glucose, blue arrows indicate unique peaks of gluconic acid, and orange arrows indicate the peaks of SP (2.3 ppm) and acetate (2 ppm). Total glucose consumption was achieved when a low sugar concentration was used (3), indicating a prolonged activity of GOx compared to the condition without H_2O_2 scavengers (Figure S3), probably due to the removal of H_2O_2 by the reaction with SP. When SP was completely decarboxylated before the consumption of the sugar, the enzyme was probably inhibited by the accumulation of H_2O_2, as indicated by the residual glucose in the solution.

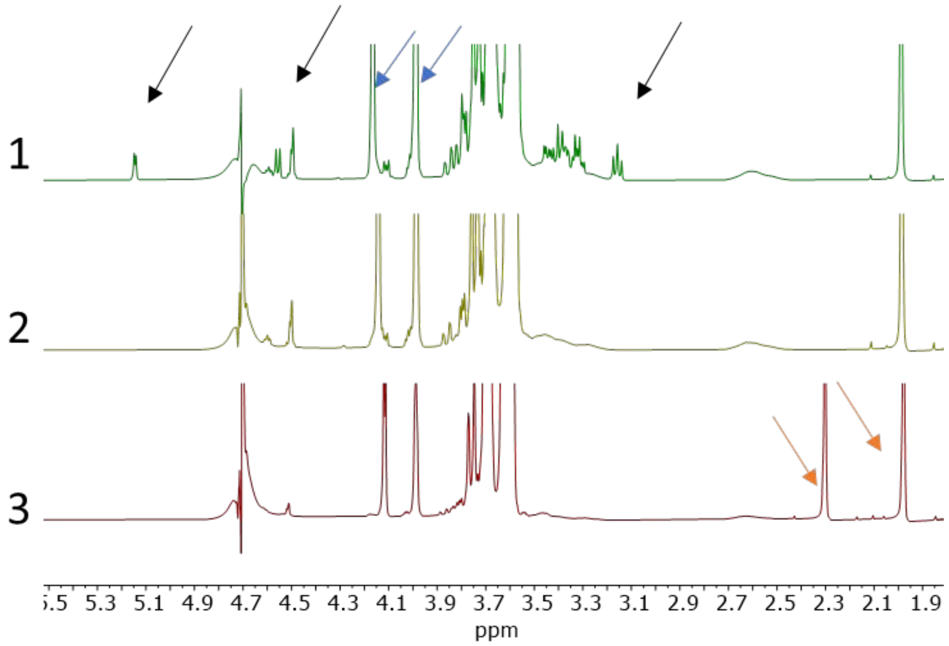

Figure S8. ^{1}H-NMR spectra of open-air reactions of GOx and NAM in the presence of different concentrations of glucose, with 400 mM SP as H_2O_2 scavenger. 1) Glucose 1 M – monomer conversion 33%; 2) Glucose 500 mM – monomer conversion 31%; 3) Glucose 200 mM – monomer conversion 20%. Black arrows indicate unique peaks of glucose, blue arrows indicate unique peaks of gluconic acid, and orange arrows indicate the peaks of SP (2.3 ppm) and acetate (2 ppm). Total consumption of glucose was achieved when low and middle concentrations of the sugar were used (2 and 3), indicating a prolonged activity of GOx compared to the condition without H_2O_2 scavengers (Figure S3) or the condition with a lower amount of H_2O_2 scavengers (Figure S4 and S5). When SP was completely decarboxylated before the consumption of the sugar, the enzyme was probably inhibited by the accumulation of H_2O_2, as indicated by the residual glucose in the solution (1).

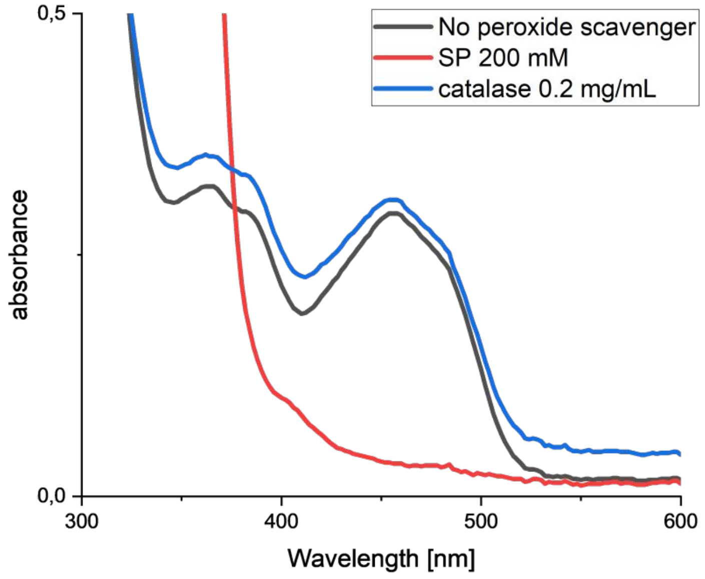

Figure S9. UV-vis spectra of reaction solutions of 5 mg/mL GOx, 200 mM glucose and 500 mM NAM without peroxide scavenger, with 200 mM SP, and with 0.2 mg/mL catalase after 18 h at 37 °C, covered with oil. The enzyme was oxidised without peroxide scavenger, and when catalase was used. When SP was used, no oxidation of GOx was observed.

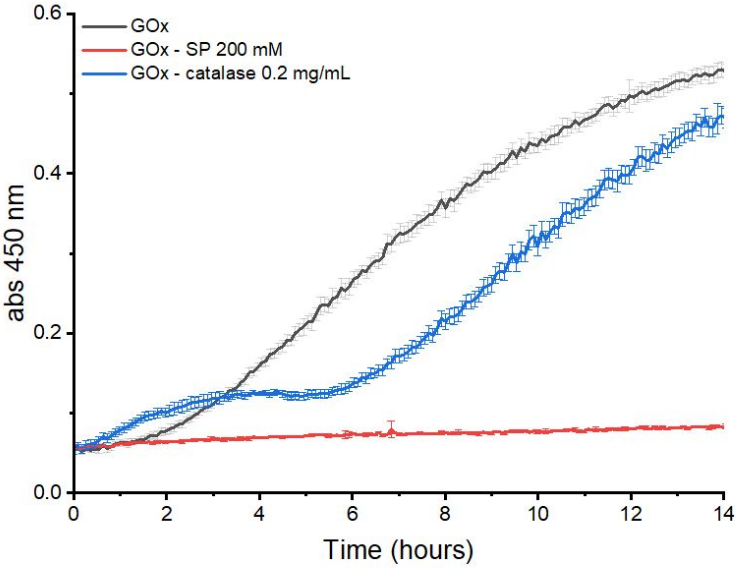

Figure S10. Reoxidation of GOx during the polymerisation of 500 mM NAM at 37°C in a low oxygen regime in the presence or absence of the peroxide scavengers catalase and SP, monitored by the absorbance of FAD at 450 nm. Catalase partially prevents the oxidation of FADH2 during the first 6 hours, while SP completely suppresses it, likely due to the efficient removal of H_2O_2 and the buffering of pH.

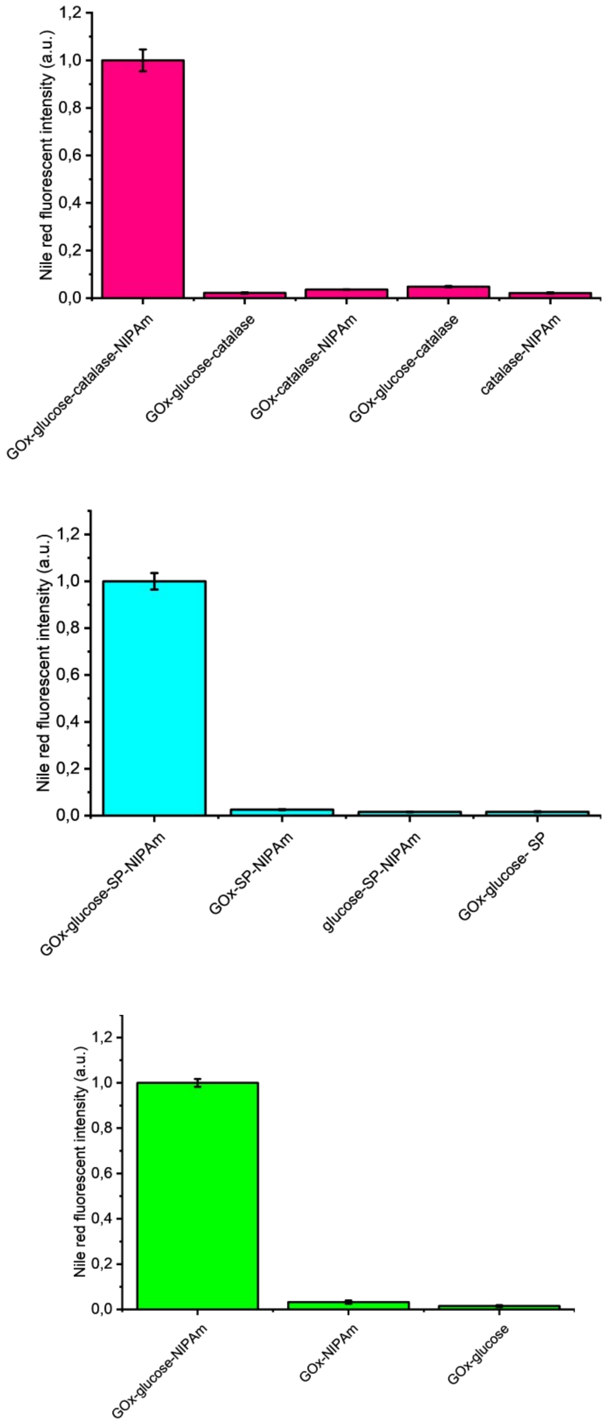

Figure S11. Nile red fluorescence emission after 18 h of reaction time. For all the reactions: 500 mM NIPAm, 5 mg/mL GOx, 200 mM glucose were used for reaction at 30 °C in a low-oxygen regime achieved by covering the reaction mixture with mineral oil. Control reactions were performed by omitting one or more of the essential components of the reaction (monomer, glucose and GOx). Magenta: 0.2 mg/mL catalase used as scavenger; cyan: SP 200 mM SP used as scavenger; green: no peroxide scavenger used.

Table S4. Nile Red fluorescence intensity and monomer conversion of GOx-initiated polymerisations of NIPAm under different conditions (GOx and NIPAm concentration) after 18 h of reaction at 30 °C. The reported values represent the average of three different measurements.

	GOX 5 mg/mL		GOX 1 mg/mL		GOX 0.5 mg/mL		GOx 0.1 mg/mL	
	NIPAm 500 mM	NIPAm 300 mM	NIPAm 500 mM	NIPAm 300 mM	NIPAm 500 mM	NIPAm 300 mM	NIPAm 500 mM	NIPAm 300 mM
Monomer conversion (%)	81	70	60	45	42	39	35	32
Nile Red fluorescence (a.u.)	98859	73489	60602	43434	34319	27999	16803	12076

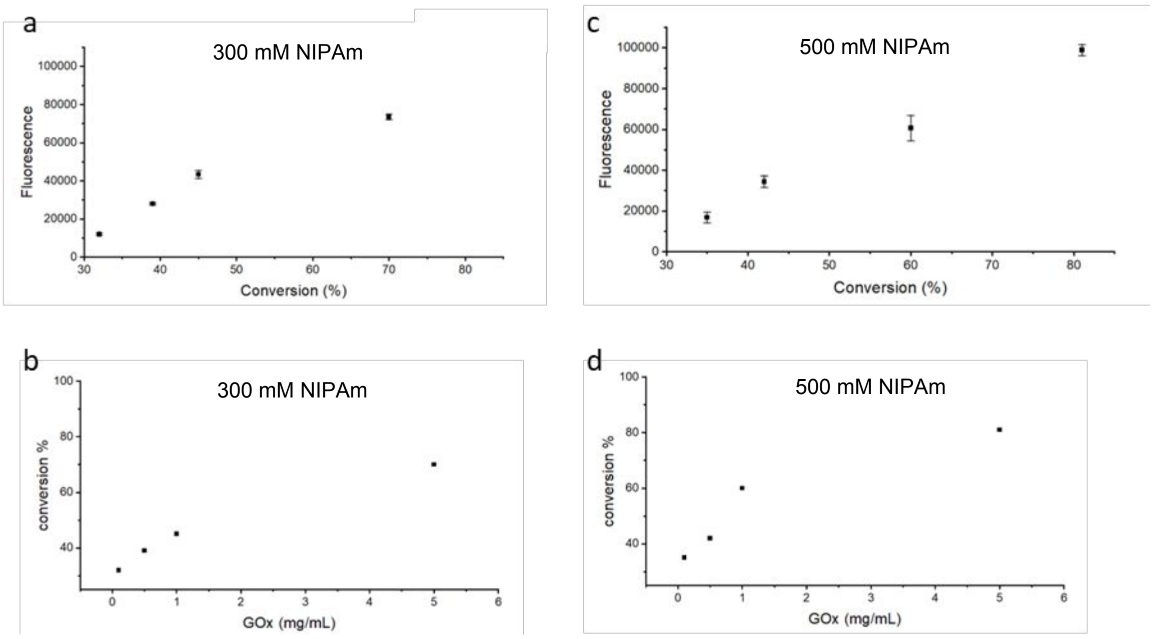

Figure S12. Nile Red fluorescence intensity and monomer conversion of GOx-initiated polymerisations of NIPAm under different conditions (GOx and NIPAm concentration) after 18 h of reaction at 30 °C. (a – b) Reaction of 300 mM NIPAm and various GOx concentrations (0.1, 0.5, 1, and 5 mg/mL). (c – d) reaction of 500 mM NIPAm and various GOx concentrations (0.1, 0.5, 1, and 5 mg/mL). For each measurement, the mean ± SD is reported; n = 3. The Nile Red fluorescence is proportional to the monomer conversion, and final monomer conversion and final Nile Red fluorescence intensity at 18 h of reaction are proportional to the amount of GOx used.

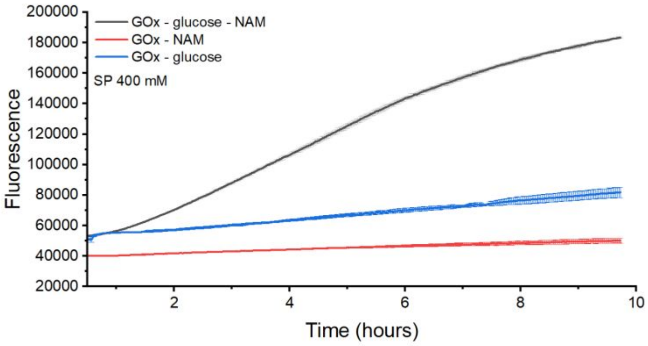

Figure S13. Free radical assay based on Dihydrorhodamine 123 (DHR) oxidation. When 500 mM NAM was added to a solution of GOx (5 mg/mL) and 200 mM glucose, Dihydrorhodamine 123 was oxidised to the fluorescent rhodamine 123. The assay was performed in the presence of 400 mM SP to ensure removal of peroxide, in a volume of 200 µL covered with heavy mineral oil. Final DHR concentration: 385 µM. Excitation: 487 nm; emission: 535 nm. The measurements were collected in a dark-clear bottom 96-well plate using a bottom optic setting. For each measurement, the mean ± SD is reported; n = 3.

Table S5. Results of the molecular docking of monomers into the active site of GOx. The results show a direct interaction between the docked molecule and the reduced cofactor of the enzyme. Dissociation constant values are calculated by YASARA and distance between the monomer and the reduced cofactor are analysed by PyMOL.

**Monomer**	**K_d (M)**	**Distance H-N5 FAD (Å)**
NAM	4.8×10^{-5}	2.1
NASS	3.78×10^{-6}	3.1
NIPAM	2.3×10^{-4}	2.2
VP	1.0×10^{-4}	3.6
AN	3.87×10^{-3}	3.2

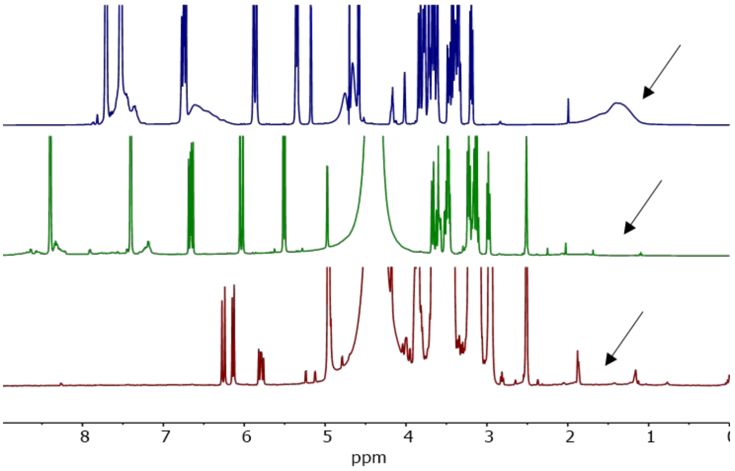

Figure S14. ^{1}H NMR spectra of GOx-initiated polymerisations of 500 mM NaSS in D_2O (blue), 500 mM 4VP in DMSO-d_6 (green), and 700 mM AN in DMSO-d_6 (red). The reactions were performed in anoxic conditions with 5 mg/mL GOx, and 200 mM glucose for 18 h at 37°C in an argon atmosphere. Black arrows indicate the expected position of the polymer backbone signal.

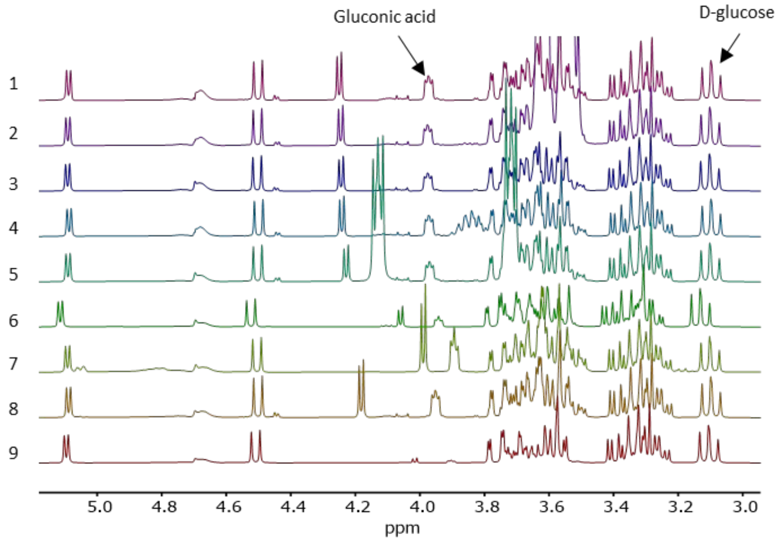

Figure S15. ^{1}H-NMR spectra to determine the conversion of glucose to gluconic acid by GOx under different conditions. For all conditions tested: 5 mg/mL GOx, 200 mM glucose in 50 mM PB pH 6, 37 °C, overnight, monomer concentration: 500 mM. 1) No monomer added; 2) NAM, 3) AAm, 4) NIPAM, 5) HEMA, 6) NaSS, 7) 44VP, 8) AN, 9) Heat-denatured GOx. The shift in gluconic acid peaks is due to differences in the pH. Black arrows indicate the peaks considered for the activity measurements.

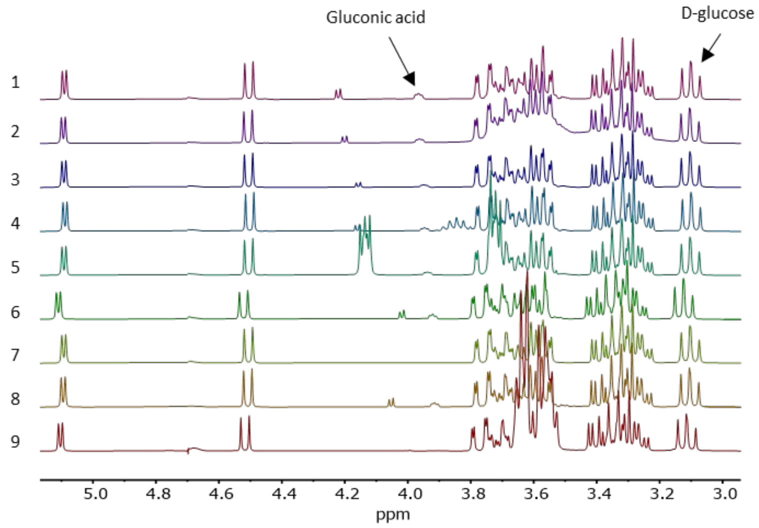

Figure S16. ^{1}H-NMR spectra to determine the conversion of glucose to gluconic acid by GOx under different conditions. GOx was incubated overnight at 37 °C with the monomers before 200 mM glucose was added to the mix. For all conditions tested: 5 mg/mL GOx in 50 mM PB pH 6, 37 °C, overnight, monomer concentration: 500 mM. 1) No monomer added; 2) NAM, 3) AAm, 4) NIPAM, 5) HEMA, 6) NaSS, 7) 44VP, 8) AN, 9) Heat-denatured GOx. The shift in gluconic acid peaks is due to differences in the pH. Black arrows indicate the peaks considered for the activity measurements.

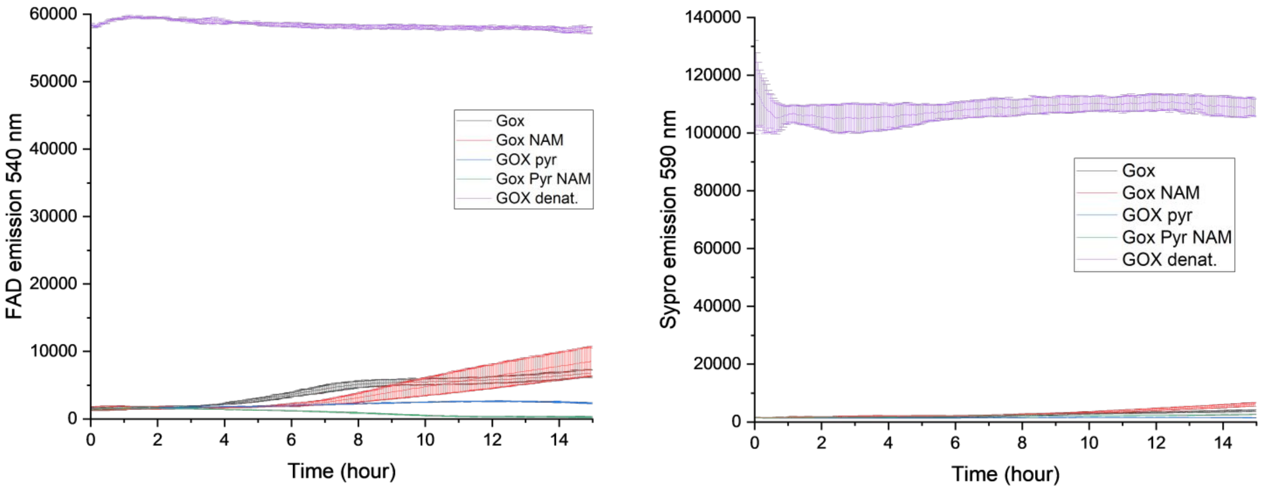

Figure S17. Stability of GOx during the GOx-initiated polymerisation of 500 mM NAM in a low oxygen regime, measured by the absorbance of the FAD cofactor and by fluorescence of Sypro orange. The release of the cofactor (top) was followed by fluorescent emission of FAD in solution (ex. 450 nm; em: 540 nm). Sypro Orange (ex: 490 nm; em: 590 nm) was used to determine the folding state of the protein. For each measurement, the mean ± SD is reported; n = 3.
